# Mycorrhizal Stimulation of Leaf Gas Exchange in Relation to Root Colonization, Shoot Size, Leaf Phosphorus and Nitrogen: A Quantitative Analysis of the Literature Using Meta-Regression

**DOI:** 10.3389/fpls.2016.01084

**Published:** 2016-07-29

**Authors:** Robert M. Augé, Heather D. Toler, Arnold M. Saxton

**Affiliations:** ^1^Department of Plant Sciences, University of Tennessee, KnoxvilleTN, USA; ^2^Department of Animal Science, University of Tennessee, KnoxvilleTN, USA

**Keywords:** arbuscular mycorrhiza, carbon exchange rate, leaf nutrient concentration, meta-analysis, photosynthesis, stomatal conductance, transpiration

## Abstract

Arbuscular mycorrhizal (AM) symbiosis often stimulates gas exchange rates of the host plant. This may relate to mycorrhizal effects on host nutrition and growth rate, or the influence may occur independently of these. Using meta-regression, we tested the strength of the relationship between AM-induced increases in gas exchange, and AM size and leaf mineral effects across the literature. With only a few exceptions, AM stimulation of carbon exchange rate (CER), stomatal conductance (*g*_s_), and transpiration rate (*E*) has been significantly associated with mycorrhizal stimulation of shoot dry weight, leaf phosphorus, leaf nitrogen:phosphorus ratio, and percent root colonization. The sizeable mycorrhizal stimulation of CER, by 49% over all studies, has been about twice as large as the mycorrhizal stimulation of *g*_s_ and *E* (28 and 26%, respectively). CER has been over twice as sensitive as *g*_s_ and four times as sensitive as *E* to mycorrhizal colonization rates. The AM-induced stimulation of CER increased by 19% with each AM-induced doubling of shoot size; the AM effect was about half as large for *g*_s_ and *E.* The ratio of leaf N to leaf P has been more closely associated with mycorrhizal influence on leaf gas exchange than leaf P alone. The mycorrhizal influence on CER has declined markedly over the 35 years of published investigations.

## Introduction

Arbuscular mycorrhizal (AM) symbiosis often modifies gas exchange of the host plant (e.g., Koide, 1993; Smith and Read, 2008; Ruiz-Lozano and Aroca, 2010; Augé et al., 2014a). Photosynthetic rates, stomatal conductance (*g*_s_), and transpiration rates (*E*) are often higher in AM plants relative to their non-mycorrhizal (NM) controls, although sometimes no effect or negative effects have been reported. Explanations for how the symbiosis might bring about these changes include redistribution or more effective scavenging of soil water, altered root aquaporins and hydraulic conductance, modified chemical root-to-shoot signaling, and changes in plant and soil water potential components and their soil-to-root gradients (Augé, 2001; Augé et al., 2004; Allen, 2007; Xu et al., 2013).

Since the earliest reports of AM-induced changes in plant–water relations and stomatal behavior, it has been suggested that at least some of the mycorrhizal effects are related to enhanced nutrition and associated effects on shoot growth (Augé, 2001). Physiological processes may be more robust in better nourished plants, perhaps especially during stress, and larger plants may display different gas exchange rates than smaller plants, regardless of mycorrhization of roots. Leaf phosphorus concentration (referred to subsequently as leaf P) can regulate photosynthesis (Walker et al., 2014) and affect stomatal behavior (Atkinson and Davison, 1972; Nagarajah and Ratnasuriya, 1978). Koide (1985) and Fitter (1988) demonstrated strong positive relationships between *g*_s_ and leaf P and suggested that AM effects on *g*_s_ in those experiments were tied to P nutrition. Others have not observed these relationships or have observed AM effects on gas exchange that go beyond nutrition (Augé, 2001). Hoeksema et al. (2010) have shown that interactions between tissue nutrient concentrations, particularly N:P ratios, may better predict plant response to mycorrhizae than leaf P.

Investigators have sometimes observed significant correlations between a water relations or gas exchange parameter and extent of root colonization. For example, soil moisture content at permanent wilting of individual plants was closely inversely correlated with root colonization (Bethlenfalvay et al., 1988), and AM-induced increases in carbon exchange rate (CER), *g*_s_, and *E* were related to the degree of mycorrhizal colonization (Abdel-Fattah et al., 2014). Changes in percent root colonization have also been positively associated with changes in shoot K^+^/Na^+^ (Augé et al., 2014b). However, AM effects on host physiology have often not been well-correlated with percent root colonization (e.g., Dakessian et al., 1986; Fitter and Merryweather, 1992; Ruiz-Lozano et al., 1995).

Our objective was to determine if, across the literature, AM-induced changes in host gas exchange are correlated with the AM-induced changes in size and nutrition. We also included extent of root colonization because it can be expected to be at least loosely tied to the size of the AM effect on nutrient absorption and host growth. Although mycorrhizal effects on host physiology have long been suspected to result in part from mycorrhizal effects on plant size and tissue nutrient concentrations, the majority of gas exchange studies have not reported correlation tests for *g*_s_, CER, and *E* with the individual plant’s leaf P or shoot size. Typically, though, AM and NM treatment means for these parameters have been reported. The value of meta-analysis is that it allows the means of each primary study to act as replicates in much the same way that individual plants or plots serve as treatment replicates in primary studies. Weighted average relationships can be computed across studies to test for treatment effects, and regression analysis can test for covariates or factors that might explain the effect. In meta-analysis, these covariates are termed explanatory variables, moderating variables, or simply, moderators. Averaging or regressing across studies has the limitation that experiments were performed under non-standardized, widely varying experimental conditions. However, this can also be an advantage; we can investigate whether a mycorrhizal effect has been more pronounced or consistent under some experimental conditions than others.

We sought to answer these questions using all available data in the literature:

(1) Has the extent of AM influence on CER, *g*_s_, and *E* been positively related to the extent of root colonization?
(2) Has the extent of AM influence on *g*_s_, CER, and *E* been related to the size of AM-induced impacts on shoot growth and leaf P?
(3) Is leaf N:P a better explanatory covariate than leaf P, for gas exchange parameters?

We focused on these questions because they have been central in attempting to understand how the symbiosis stimulates gas exchange. Many investigators have tested whether the degree of mycorrhizal influence can be related to the extent to which root systems are colonized by these fungi (Smith and Read, 2008). There are limitations in using one-time measurements of final, static colonization rates to portray how thoroughly a symbiont may be associated with and influencing the physiology of the other symbiont, but this is what is commonly reported. With our first question above we sought to test the strength (or lack thereof) of the association between root colonization rates and the three measures of gas exchange with the greatly increased statistical power of meta-analysis. Other common measures of AM influence on plants involve nutrition and size. Since the first reports of AM influence on the water relations of the host plant, AM-induced enhancement of P nutrition and rate of plant growth have been implicated in the AM effect (Safir et al., 1971, 1972; Liu et al., 2007). Many subsequent experiments over the years provide evidence that tends to confirm or refute this (Augé, 2001; Ruiz-Lozano and Aroca, 2010). Examining each AM-induced change in gas exchange parameters in light of AM-induced changes in plant size and nutrition offers an integrated view of the association across the literature. We tested the N:P ratio as well as P alone, as others have suggested that the relative abundance of P in relation to N is a better predictor of AM influence than P alone (Hoeksema et al., 2010).

## Materials and Methods

### Data Collection

Using the ISI Web of Science search tool (Thompson Reuters Corp., Toronto, ON, Canada), studies were identified through a systematic search of 12 electronic databases for refereed and non-refereed articles. Articles were found in CAB International, Biosis Citation Index, Web of Science Core Collection, Biological Abstracts, Current Contents Connect. On 30 January 2015, a search through the year 2014 using the following terms gave 1266 search results: (mycorrhiz^∗^ OR endomycorrhiz^∗^ OR “AM fungi” OR “AM symbiosis” OR “VAM fungi” OR “VAM symbiosis”) AND (“stomatal conductance” OR “stomatal resistance” OR transpiration^∗^ OR “gas exchange rate” OR “photosynthetic rate” OR “carbon exchange rate” OR “C exchange rate” OR “CO_2_ exchange rate” OR “carbon assimilation rate” OR “C assimilation rate” OR “CO_2_ assimilation rate”). An additional 15 articles were found in article bibliographies. Papers unavailable online were requested from the University of Tennessee Interlibrary Loan Service. We did not attempt to obtain dissertations. With examination of these 1281 eligible articles, 1056 were excluded because they did not meet the following inclusion criteria: *g*_s_, CER, or *E* were not reported; AM or NM treatments were not included; uninoculated NM controls were mycorrhizal with mean root colonization of ≥5%; mean root colonization of AM plants was 0%; article was a duplicate; article did not contain primary data (review or book). We were unable to obtain five articles. We identified 220 journal articles and conference proceedings that met our screening criteria (details of primary studies provided in **Supplementary Material, Data Sheet S1**). Papers spanned 35 years and were in English, Chinese, Spanish, Portuguese, French, and Persian.

Treatment means and sample sizes were collected for each study. If sample size was given as a range, we used the smallest value. For studies that did not report sample size, we used *n* = 1 (4 articles, 17 studies) unless least significance difference (LSD) or standard errors were provided, in which case we used *n* = 2 (7 articles, 25 studies). Including these studies increased the power of the analysis, with their weight limited by a conservative assignment of sample size. If data were provided in graphical form, means were extracted using WebPlotDigitizer (Rogatgi, 2011).

Multiple treatments or host/symbiont combinations from one article were treated as independent studies (sometimes referred to as paired observations in the meta-analysis literature) and represented an individual unit in the meta-analyses. For example, Fay et al. (1996) provided gas exchange data for AM and NM treatments given five different phosphorus treatments, resulting in five studies from that article. Although designating multiple studies from one publication has the disadvantage of increasing the dependence among studies that for the purposes of meta-analysis are assumed to be independent (Gurevitch and Hedges, 1999), the greater number of studies increases statistical power (Lajeunesse and Forbes, 2003). This approach has been used commonly in mycorrhizal and plant biology meta-analyses (e.g., Hoeksema et al., 2010; Holmgren et al., 2012; Veresoglou et al., 2012; Mayerhofer et al., 2013; Chandrasekaran et al., 2014). We derived 1019 studies from the 220 articles. Plant hosts were represented by 121 species and 97 genera, and fungal symbionts by 30 species and 9 genera.

### Effect Sizes and Moderator Variables

We conducted meta-analysis and meta-regression on three leaf gas exchange characteristics: CER, *g*_s_, and *E*. Studies were evaluated via treatment effect size (ES), which was computed as the natural logarithm of the response ratio (ln*R*) of the mycorrhizal to NM means:

$$InR=InY_{AM}/Y_{NM}$$

where *Y*_AM_ and *Y*_NM_ are means of AM treatments and NM controls. For meta-analysis, these were used to measure the overall, “global” effect: the summary or cumulative AM/NM ES across studies (Borenstein et al., 2009). For meta-regression, study ESs were used to assess relationships between gas exchange and quantitative independent variables (regression moderators). It is common to use a response ratio in meta-analyses of plant and mycorrhizal behaviors (e.g., Lehmann et al., 2012; Mayerhofer et al., 2013; Jayne and Quigley, 2014), as it gives a standardized, unit-less expression of treatment-induced change. The log transformation is needed to properly balance positive and negative treatment effects across response ratios (to maintain symmetry in the analysis). Stomatal resistance values (*r*_s_; inverse of *g*_s_) were converted to *g*_s_. If photosynthetic or transpiration rates were reported on a whole plant basis and leaf area data were provided, we calculated CER and *E* for AM and NM treatments (e.g., Jia and Gray, 2008). Gas exchange measurements during the dark were not included.

Values of 0 are biologically common but mathematically not possible to incorporate into meta-analysis (ratio denominator cannot be 0; cannot take the natural log of 0). A common technique used in medical literature is to add a small fixed number to any zero value (NCSS Statistical Software, 2015). In gas exchange research, however, this technique yields very inconsistent results, owing to the wide variety of units and the wide range of maximal gas exchange values. Further, small non-zero values result in unreasonably inflated response ratios. In order to analyze gas exchange ESs of zero and near zero, we calculated 1% of the highest gas exchange value for a study and raised any other value below 1% to that level: for example, to 2.5 for 250 mmol m^-2^ s^-1^, to 0.03 for 3.0 mm s^-1^. Negative values of gas exchange were equated to 0 before applying the 1% adjustment. The adjustment was made to two of the 583 CER studies and one of the 704 *g*_s_ studies.

In addition to the three gas exchange measures for computing ES, we recorded information for five quantitative, physiological moderators: root colonization percentage, shoot dry weight (DW), leaf P, and two forms of leaf N:leaf P ratio. We recorded the leaf N:P ratio of NM plants (NM leaf N:P), as well as the AM leaf N:P/NM leaf N:P response ratio (leaf N:P ES). The association of the quantitative moderators with gas exchange was examined with meta-regression. We also included environmental stress as a categorical moderator in multi-factor meta-regression of quantitative moderators. For studies having stress treatments, unstressed control treatments were coded as “unstressed” and stress treatments coded as “stressed.” The “stressed” level of this moderator included drought stress, salt stress, flooding stress, heat stress, cold stress, disease stress, heavy metal stress, and oxidative stress. In studies without a control group of unstressed plants, gas exchange means obtained on the last day before plants were subjected to the stress treatment were considered “unstressed.” Studies without stress treatments, and whose methods and results sections did not give evidence that plants were stressed in some way other than NM controls by P stress, were coded “unstressed.”

Where moderator data were given for more than one time period, final shoot DW, leaf P, leaf N, and root colonization percentages were used in the analyses. In a few instances, we recorded leaf area as a proxy for shoot biomass if leaf area was reported but biomass was not. When mineral concentrations or DWs were provided for whole plants and not for shoots, whole plant values were used in the analysis as reasonable proxies for the AM/NM shoot ES. When arbuscular, vesicular, and hyphal colonization rates were reported separately, we recorded the highest values. When total colonization rates were reported at different depths for the same plants, we recorded the highest values. Where colonization rate was reported as a relatively narrow range (≤25%) rather than a mean, we used the midpoint of the range for the meta-regression; for example, where root colonization was reported as 30–40%, 35% was used for the meta-regression (Brown et al., 1988).

Possible temporal changes in ES were evaluated using publication year as a sixth quantitative moderator (Koricheva and Gurevitch, 2014). We also examined time as a categorical moderator, arbitrarily breaking the 35-year span over which data were published into seven equal 5-year periods.

### Meta-Analysis and Meta-Regression

Our analyses followed the methodology and terminology of Borenstein et al. (2009) and were guided by the criteria suggested by Koricheva and Gurevitch (2014). We used a random-effects model for the meta-analyses, considering that true effects are likely to have varied across studies (rather than a fixed-model, which assumes the same value or true effect for all studies). The random-effects model was also appropriate for the meta-regression analyses, as it is plausible to think that the regression moderators (covariates) may have captured some but not all true variation among effects. We computed the CER, *g*_s_, and *E* summary effects with Comprehensive Meta-Analysis (CMA) software (Version 3, Biostat, Englewood, NJ, USA; 2014). Meta-regression analysis was also conducted with CMA, with the restricted maximum likelihood and Knapp–Hartung methods (IntHout et al., 2014).

Meta-regression produces intercept and slope estimates, where the intercept is the summary ES when the moderator is 0 and the slope is the change in ES per one unit increase in the moderator. The meta-regression *p*-value tests if this slope is equal to 0. Regressions were performed using ln*R* values. Raw, average slope over the range of *x*-axis values was computed as:

$$slope=\frac{\left[\exp({slope}_{InR,\;\max X}+{intercept}_{InR})-\exp\left({slope}_{InR,\;\min X}+{intercept}_{InR}\right)\right]}{\left(\max X-\min X\right)}$$

where max*X* and min*X* are the maximum and minimum values for the *x*-axis moderator, and exp refers to exponential function. Regression analyses were checked in CMA for influential points using Cook’s D (Viechtbauera and Cheung, 2010).

The *p*-values in the single moderator regression analyses portray the relative importance of the regression moderators. Another test of their relative importance was performed with mutlifactor regression using the smaller subset of studies that contained data for each of the five regression moderators. Here, *p*_test_ denotes “test of change” in incremental regression analysis, testing if the moderator explains significant further variation after variation by the other four moderators has been accounted for.

#### Variance

Individual studies were weighted using non-parametric variance:

$$V=\frac{\left(n_{AM}+n_{NM}\right)}{n_{AM}\times n_{NM}}$$

where *V* is the variance of the natural log of the AM/NM response ratio and *n*_AM_ and *n*_NM_ are the samples sizes of the AM and NM treatments (Rosenberg et al., 2000). Several publications did not report standard errors or standard deviations, nor was sufficient information given in many instances to estimate these from LSD or other mean separation test values. As has often been noted (e.g., Adams et al., 1997; Lehmann et al., 2012; Veresoglou et al., 2012), it is not uncommon for measures of dispersion to have been omitted from publications involving plants, which makes calculating weighting based solely on sample size (non-parametric variance) a necessity. Excluding studies that report sample size but not some measure of dispersion would represent a substantial loss of analytical power.

We did not consider measurements at multiple times as separate studies. Rather, we computed the means of the time-points and used this synthetic score as the unit of analysis as recommended for multiple time-points by Borenstein et al. (2009). The multiple time-point variance *V_y_* was computed as:

$$V_{y}=\frac{V\left(1+(m-1)r\right)}{m}$$

where *m* is the number of time-points and *r* is the correlation among time-points. The coefficient *r* describes the extent to which time-point values co-vary. If values for time-points are perfectly linked (a change in one completely describes the change in the others), then *r* = 1 and the weighting for meta-analysis is mathematically the same as a single time-point. If values for time-points are unrelated, then *r* = 0 and the variance is defined by the total sample size of the measurements. Correlations among gas exchange measurements made on individual experimental units are generally not reported. To get a sense of *r* for gas exchange, we computed it using data from Augé (2003) and Augé et al. (2008), representing herbaceous and woody genera, greenhouse and field conditions, and young and mature plants. For squash *g*_s_, *r* was 0.07. For tulip poplar *g*_s_, CER, and *E*, *r* was 0.01, 0.00, and 0.04, respectively (effectively 0). Based on these tests, we applied *r* = 0.1 for all studies in the meta-analyses. A value close to 0 makes sense biologically, as *g*_s_ and gas exchange measures related to *g*_s_ (CER, *E*) may be expected to be largely independent, given their sensitivity to environmental changes, especially when expressed on a s^-1^ basis (Augé, 2000). In the 252 studies reporting multiple time-points, sample size did not differ between AM and NM treatments, hence AM and NM non-parametric variances were equal, each represented by *V* in Eq. 4. Light response curves were treated as multiple outcomes—handled mathematically like multiple time-points (Borenstein et al., 2009)—when gas exchange measurements were made on the same plants at different light levels as is typically done (e.g., Chen et al., 2014). Multiple time-point calculations are shown in **Supplementary Material (Data Sheet S1**). Weighted correlation analysis among regression moderators was performed to assess their dependence (SAS 9.4, Cary, NC, USA).

#### Heterogeneity

Heterogeneity was assessed with the *Q* statistic (a measure of weighted squared deviations) and quantified using *I*^2^, a descriptive index that estimates the ratio of true variation (heterogeneity) to total variation across the observed ESs (Higgins and Thompson, 2002; Huedo-Medina et al., 2006). *I*^2^ is defined as (*Q*_total_ - *df*) × 100/*Q*_total_, where *Q*_total_ is total variation; degrees of freedom (*df*) represents expected, within-study variation; and *Q*_total_ - *df* is true heterogeneity, or between-study variation (*Q*_between_). A value of 0% indicates no true heterogeneity, positive values indicate true heterogeneity in the data set with larger values reflecting a larger proportion of the observed variation due to true heterogeneity among studies. Assumptions of homogeneity were considered invalid when *p*-values for the *Q*-test (*p*_hetero_) for heterogeneity were less than 0.1 (e.g., Bristow et al., 2013; Iacovelli et al., 2014). We assumed a common among-study variance across moderator subgroups.

For meta-regression, *R*^2^ analog characterizes the true variance explained as a proportion of the total true variance, defined as (Borenstein et al., 2009):

$$R^{2}\mathrm{analog}=\frac{T^{2}\,\;\exp lained}{T^{2}\,\;total}\times 100$$

where *T*^2^_explained_ is the true variance explained by the regression and *T*^2^_total_ is the total true variance.

#### Publication Bias and Sensitivity Analysis

Potential publication bias was assessed statistically with Begg and Mazumdar rank (Kendall) correlation and represented graphically with funnel plots of ESs vs. their standard errors (Begg and Mazumdar, 1994; Borenstein et al., 2009). Sensitivity analysis was performed for the global summary effects by removing one study and re-running the meta-analysis, for every study in the analysis. This shows how much each study contributed to the summary effect, by noting how much the summary effect changes in its absence.

## Results

### Meta-Analysis and Diagnostics

**FIGURE 1:**
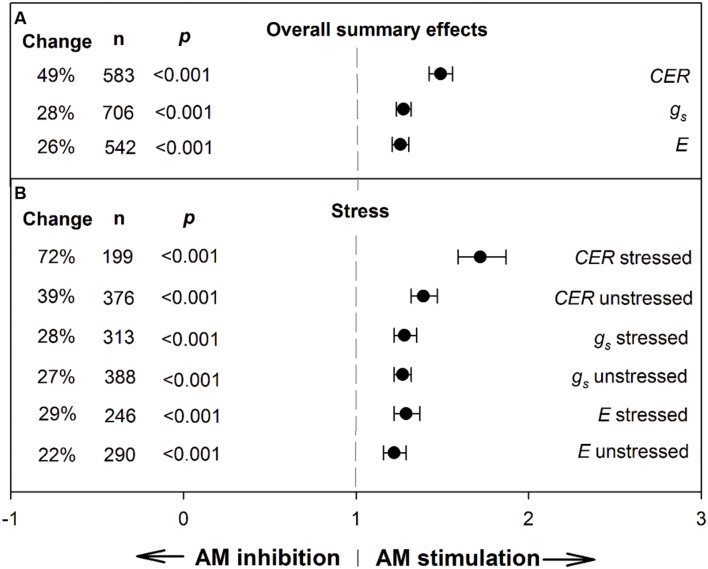
**Weighted summary effect sizes (response ratios) and 95% confidence intervals for AM influence on foliar carbon exchange rate (CER), stomatal conductance (*g*_s_), and transpiration rate (*E*).** **(A)** Overall summary effects. **(B)** Summary effects for stress moderator. Change refers to raw percentage increase in the gas exchange parameter induced by AM symbiosis. *n* is number of studies contributing to the effect size. *p* ≤ 0.05 indicates that the moderator level was significantly different than 0.

To provide context for the meta-regression analysis and derive an overall view of AM influence on the three gas exchange parameters, we conducted meta-analyses to determine summary effects (**Figure 1**; **Table 1**). The AM/NM summary response ratio for CER was 1.49 (ln*R* = 0.399); over the 583 studies having AM and NM CER means, the weighted average AM stimulation of CER was a sizeable 49% (**Figure 1**). AM effects on CER were about twice as large as AM effects on *g*_s_ and *E*. AM-induced stimulation of *g*_s_ and *E* were nearly identical and averaged 28 and 26%, respectively, over the 706 *g*_s_ studies and 542 *E* studies. The CER ES was nearly twice as large in plants exposed to stress treatments (72%) as in unstressed controls (39%). Stress did not modify the size of the AM influence on *g*_s_ or *E*.

**Table 1:** Categorical meta-analysis of three gas exchange summary effects.

Moderator	*Q*_between_	*n*	*df*	*I*^2^ (%)	*p*_hetero_
**Carbon exchange rate (CER)**					
Stress	18.9	575	1	13.4	<0.001
Chronology	54.2	583	6	12.3	<0.001
**Stomatal conductance *(*g*_s_)***					
Stress	0.1	701	1	7.9	0.714
Chronology	21.2	706	6	7.5	0.002
**Transpiration rate *(E)***					
Stress	2.0	536	1	0.0	0.161
Chronology	25.4	542	6	0.0	<0.001


**FIGURE 2:**
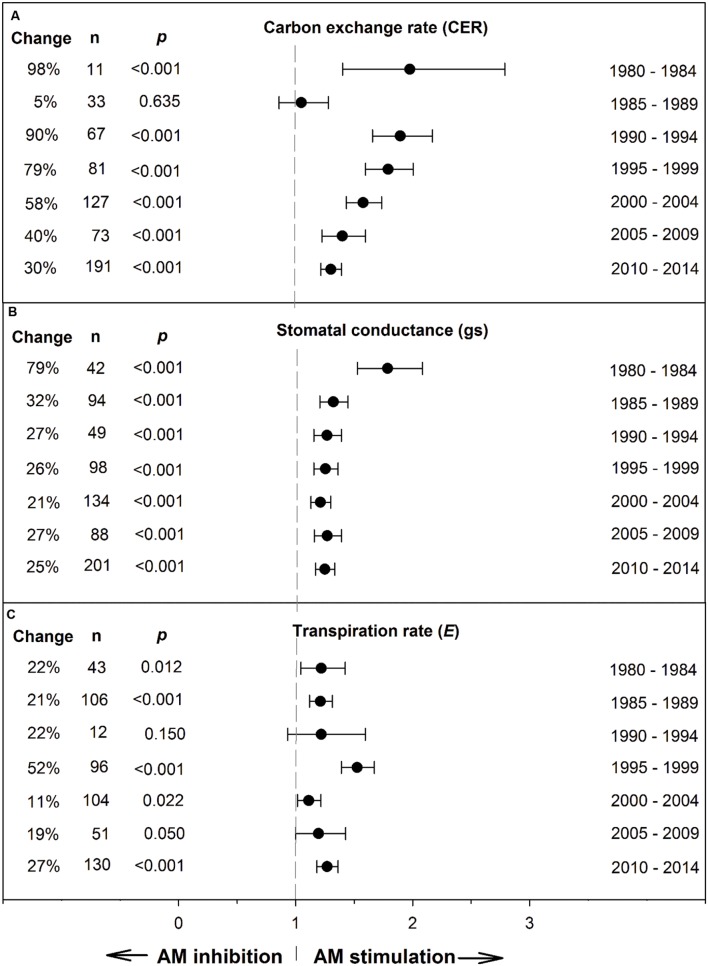
**Weighted summary effect sizes (response ratios) and 95% confidence intervals for AM influence on foliar carbon exchange rate (CER) **(A)**, stomatal conductance (*g*_s_) **(B)**, and transpiration rate (*E*) **(C)** over time (chronology moderator).** Change refers to raw percentage increase in the gas exchange parameter induced by AM symbiosis. *n* is number of studies contributing to the effect size. *p* ≤ 0.05 indicates that the moderator level was significantly different than 0.

**Q*_*between*_, between-study variation (true heterogeneity); *n*, number of studies; *df*, degrees of freedom, levels within a moderator; *I*^*2*^, the ratio of true variation (heterogeneity) to total variation; *p*_*hetero*_, p-value that all observed (total) variation is due to sampling error (within-study variation). CER, *g*_*s*_, and *E* are AM/NM effect sizes; analysis was conducted on log-transformed values (ln*R*) from each study. The levels of each categorical moderator with their summary effect sizes, confidence intervals, AM-induced change as a percentage, number of studies, and significance values are given in **Figures 1** and **2.***

We did not see evidence of publication bias. Visually, the funnel plots for each of the summary effects showed no pattern that would reflect bias toward not reporting small or negative ESs. Large or small studies across the range of standard errors had the expected variability around the common ES. Within the Begg and Mazumdar rank correlation test, each of the summary effects had absolute Kendall tau values below 0.13, indicating no publication bias (no tendency for ESs to increase as study size decreases).

The stability of the summary effects was assessed with sensitivity analysis. One study was removed and the summary effect recalculated, and this was repeated for all studies to determine how much any one study affected the summary ES. The study with the largest influence on CER was study 482, AM/NM ES 24.6 (ln*R* = 3.203, drought treatment; Amerian et al., 2001; **Supplementary Material, Data Sheet S1**), whose removal changed the summary effect by 1.7% (from 1.49 to 1.47). The study with the largest influence on *g*_s_ was study 182, ES 6.84 (ln*R* = 1.923, heat treatment; Newman and Davies, 1988; **Supplementary Material, Data Sheet S1**), whose removal changed the summary effect by 0.6% (from 1.28 to 1.27). The study with the largest influence on *E* was study 177, ES 4.81 (ln*R* = 1.57, heat treatment; Newman and Davies, 1988; **Supplementary Material, Data Sheet S1**), whose removal changed the summary effect by 0.9% (from 1.26 to 1.25). No one study changed any of the three gas exchange summary effects very much, due in large part to the large numbers of studies. Each of the most extreme studies noted above were the end points of a continuum; the next most extreme study was within 0.02 ln*R* for CER and within 0.01 ln*R* for *g*_s_ and *E*.

Koricheva and Gurevitch (2014) recommended testing whether a summary effect has changed over time, when studies comprising the effect have been published over many years. Changes in the summary effect could potentially result from publication bias, changes in methodology, or real biological changes. Investigating chronology (year of publication), as a categorical moderator consisting of seven 5-year periods, revealed that the mycorrhizal influence on CER clearly declined during the 35-year time span, with the size of the AM stimulation dropping from 98% during 1980–1985 to 30% during 2010–2014 (**Figure 2**; **Table 1**). The AM/NM summary effect on *g*_s_ was quite high (79%) during the first time period and consistently 20–30% over the subsequent six time periods. AM influence on *E* was fairly stable over the 35 years, about 20% for four of the seven time periods. The jump to 52% in 1995–1999 was attributable to relatively large summary effects in two series of stress experiments (Ruiz-Lozano et al., 1995, 1996) that accounted for 46 of the 96 studies in this 5-year period.

### Meta-Regression

#### Single Factor Meta-Regression

The shoot DW, leaf P, and AM/NM leaf N:P moderators are ESs; each is the AM/NM response ratio and represents a direct, relative measure of the AM-induced impact on the parameter. For example, a value of 2.0 for the shoot DW ES indicates that AM plants had twice the shoot DW of NM plants in that study; a value of 0.8 for leaf P signifies that AM plants had 20% less leaf P than their NM counterparts. Correlation between regression moderators is shown in **Table 3.** Six of the ten correlations were significant but *r* coefficients were generally low. Among the nutritional ES moderators, leaf N:P ES showed the strongest correlation with root colonization and shoot DW ES.

Almost all of the regressions of the gas exchange response ratios with the quantitative moderators had slopes significantly different than 0 (*p* < 0.05; **Table 2**). The moderators were better predictors of AM-induced stimulation of CER and *g*_s_ than of *E*. The CER and *g*_s_ ESs were positively associated with root colonization, leaf N:P ratio of NM plants, and with AM-induced increases in shoot DW and leaf P. AM-induced increases in CER and *g*_s_ were negatively associated with the leaf N:P ES (AM leaf N:P/ NM leaf N:P). The associations of *E* with root colonization, shoot DW ES, and leaf N:P ES were significant. *E* was not associated with the AM-induced changes in leaf P or leaf N:P of NM plants.

**Table 2:** Single factor meta-regression.

Moderator	*n*	Intercept	Slope	Average slope	*p*	*I*^2^ (%)	*p*_hetero_	*R*^2^ analog
**Carbon exchange rate (CER)**								
Root colonization	504	-0.0149	0.0079	0.0119	<0.001	13.2	0.010	35.8
Shoot DW ES	476	0.3007	0.0500	0.1923	<0.001	22.7	<0.001	37.7
Leaf P ES	334	0.2612	0.0713	0.2022	<0.001	8.6	0.116	97.2
NM leaf N:P	236	0.2593	0.0070	0.0136	0.011	0.0	0.935	–
Leaf N:P ES	242	1.0415	-0.7332	-0.8997	<0.001	0.0	0.941	–
Year	583	21.8340	-0.0107	-0.0171	<0.001	12.3	0.011	16.3
**Stomatal conductance *(*g*_s_)***								
Root colonization	636	0.0842	0.0035	0.0046	<0.001	0.0	0.968	17.6
Shoot DW ES	615	0.2007	0.0366	0.0944	<0.001	8.5	0.055	10.7
Leaf P ES	405	0.0753	0.1584	0.5156	<0.001	0.0	0.557	41.3
NM leaf N:P	179	0.3115	0.0016	0.0022	0.003	0.0	0.547	60.9
Leaf N:P ES	185	0.8739	-0.5936	-0.7126	<0.001	0.0	0.564	100.0
Year	706	11.8610	-0.0058	-0.0077	0.004	7.5	0.068	0.0
**Transpiration rate *(E)***								
Root colonization	455	0.1472	0.0022	0.0029	0.027	0.0	0.999	2.9
Shoot DW ES	461	0.1613	0.0369	0.0921	<0.001	0.0	0.854	7.6
Leaf P ES	294	0.2480	0.0078	0.0102	0.846	0.0	0.996	0.0
NM leaf N:P	142	0.3938	0.0004	0.0006	0.604	2.8	0.391	0.0
Leaf N:P ES	142	0.8742	-0.5256	-0.6690	<0.001	2.8	0.391	28.5
Year	542	3.2287	-0.0015	-0.0019	0.504	0.0	0.979	0.0


**FIGURE 3:**
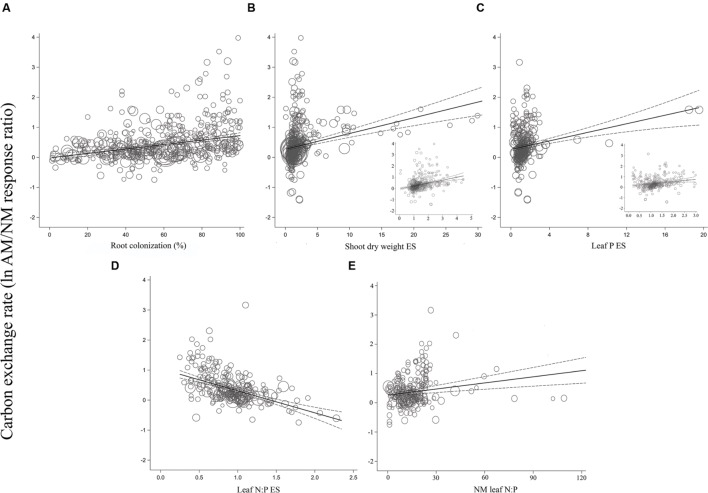
**Regression plots of the size of the AM-induced change in carbon exchange rate (ln*R* CER) as a function of **(A)** percent root colonization; **(B)** AM/NM shoot DW effect size; **(C)** AM/NM leaf P effect size; **(D)** AM/NM leaf N:P effect size; **(E)** leaf N:P of NM plants.** Darker center line is the model prediction; lighter lines are 95% confidence intervals. Each symbol represents one study, with symbol size indicating its weighting. Insets in **(B)** and **(C)** are expanded views of the ranges containing the majority of studies.

**FIGURE 4:**
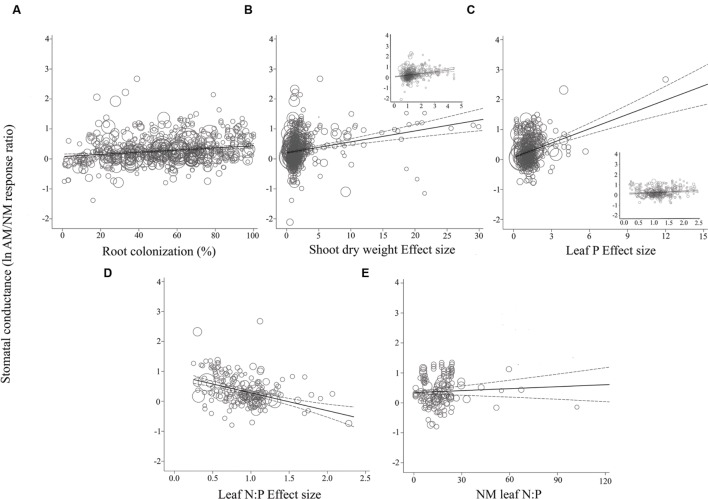
**Regression plots of the size of the AM-induced change in stomatal conductance (ln*R g*_s_) as a function of **(A)** percent root colonization; **(B)** AM/NM shoot DW effect size; **(C)** AM/NM leaf P effect size; **(D)** AM/NM leaf N:P effect size; **(E)** leaf N:P of NM plants.** Darker center line is the model prediction; lighter lines are 95% confidence intervals. Each symbol represents one study, with symbol size indicating its weighting. Insets in **(B)** and **(C)** are expanded views of the ranges containing the majority of studies.

**FIGURE 5:**
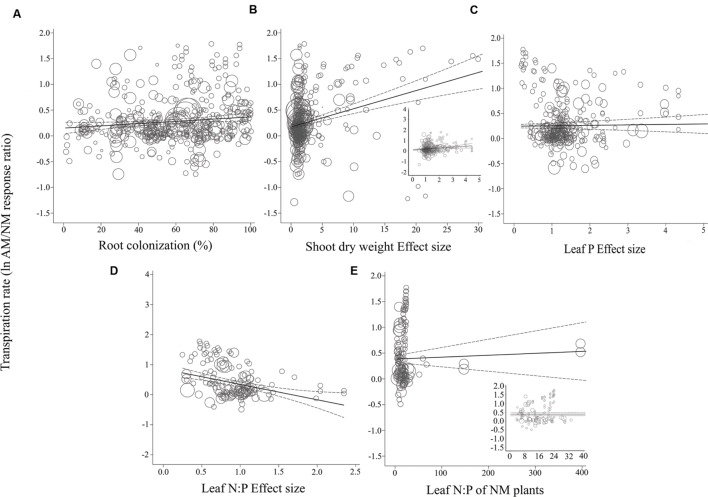
**Regression plots of the size of the AM-induced change in transpiration rate (ln*R E*) as a function of **(A)** percent root colonization; **(B)** AM/NM shoot DW effect size; **(C)** AM/NM leaf P effect size; **(D)** AM/NM leaf N:P effect size; **(E)** leaf N:P of NM plants.** Darker center line is the model prediction; lighter lines are 95% confidence intervals. Each symbol represents one study, with a symbol size indicating its weighting. Insets in **(B)** and **(E)** are expanded views of the range containing the majority of studies for those moderators.

**FIGURE 6:**
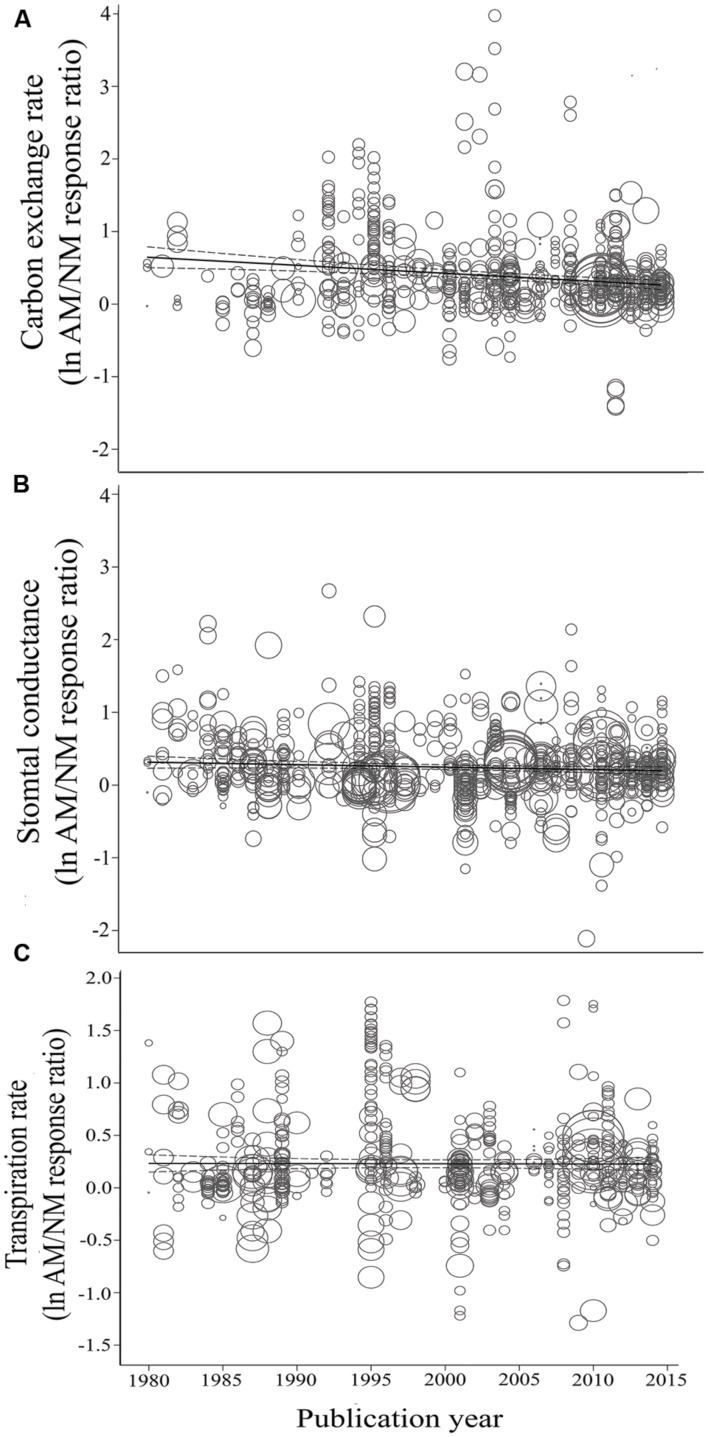
**Regression plots of the size of the AM-induced change in carbon exchange rate (CER) **(A)**, stomatal conductance (*g*_s_) **(B)**, and transpiration rate (*E*) **(C)** as a function of publication year. ln*R* is natural log of the AM/NM response ratio for each gas exchange parameter.** Darker center line is the model prediction; lighter lines are 95% confidence intervals. Each symbol represents one study, with symbol size indicating its weighting.

*CER, *g*_*s*_, and *E* are AM/NM effect sizes; analysis was conducted on log-transformed values (ln*R*) from each study. Predicted regression lines, confidence intervals and study effect sizes are depicted in **Figures 3**–**6.** ES, effect size, denotes that moderator is the study’s AM/NM effect size (response ratio) for shoot dry weight (DW), leaf P concentration, and ratio of leaf N to P concentration. NM leaf N:P is the ratio of leaf N concentration to leaf P concentration in the non-mycorrhizal control plants. Slope column gives log values; average slope column gives the average raw slopes, computed from ln slope, ln intercept, and min and max *X* values; *n*, number of studies; intercept, point at *X* = 0 where predicted model line crosses *y*-axis; *p*, probability that slope is 0; *I*^*2*^, percent of variation due to real differences among study effects; *p*_*hetero*_, probability that all observed (total) variation is due to sampling error (within-study variation). *R*^*2*^ analog, the true variance explained as a proportion of the total true variance = *T*^*2*^_*explained*_ /*T*^*2*^_*total*_ × 100 where *T*^*2*^_*explained*_ is the true variance explained by the regression and *T*^*2*^_*total*_ is the total true variance.*

**Table 3:** Correlation analysis of regression moderators.

	Colonization	Shoot DW ES	Leaf P ES	Leaf N:P ES	NM leaf N:P
Colonization		0.169^∗∗∗^	0.079	-0.319^∗∗∗^	-0.160^∗∗^
Shoot DW ES	-		0.181^∗∗∗^	-0.188^∗∗^	-0.053
Leaf P ES	-	-		-0.371^∗∗∗^	-0.047
Leaf N:P ES	-	-	-		0.055
NM Leaf N:P	-	-	-	-	


*Shown are correlation coefficients (*r*). ^∗^, ^∗∗^, and ^∗∗∗^ indicate that correlations were significant at *p* = 0.05, 0.01, or 0.001, respectively; correlation coefficients not followed by asterisk(s) indicate correlation was not significant. ES, effect size, denotes that moderator is the study’s AM/NM effect size (response ratio) for shoot dry weight (DW), leaf P concentration, and ratio of leaf N to P concentration (N:P). NM leaf N:P is the ratio of leaf N concentration to leaf P concentration in the non-mycorrhizal control plants.*

The average slopes in **Table 2** express the changes in the gas exchange parameters induced by AM symbiosis as raw percentages per unit change in the moderators, over the range of moderator values in the literature. While the CER, *g*_s_, and *E* ESs were all significantly associated with root colonization, CER was particularly sensitive, 2.6× to 4× more responsive than *g*_s_ or *E* to increases in colonization. The AM-induced stimulation of CER was increased by 1.2% with each 1% increase in colonization rate, vs. 0.5 and 0.3% for *g*_s_ and *E*, respectively. These increases are substantial. A 40% change in root colonization equates to the 49% average AM-induced increase in CER. In comparison, a 40% increase in root colonization has been associated with an AM-induced stimulation of *g*_s_ by 20%. The AM-induced stimulation of CER increased by 19% with each unit increase in shoot DW ES (i.e., with each AM-induced doubling of shoot size). The effect was about half as large for *g*_s_ and *E*, where the AM-induced stimulation rose by 9% with each unit increase in shoot DW ES. The AM-induced stimulation of CER with each unit increase in leaf P ES (each doubling of leaf P) was about the same as for AM-induced size increases; promotion of CER increased by 20% with each unit increase in leaf P. The AM-induced stimulation of *g*_s_ was markedly higher with leaf P increases than with size increases. The AM promotive effect increased by 52% with each unit increase in leaf P. Alternately, AM-induced increases in *E* were not affected by AM-induced changes in leaf P.

The CER, *g*_s_, and *E* ESs were all significantly negatively associated with leaf N:P ES (*p* < 0.05; **Table 2**). The AM-induced stimulation of CER, *g*_s_, and *E* decreased by 90, 71, and 67%, respectively, with each unit increase in AM/NM leaf N:P (i.e., with each AM-induced doubling of leaf N:P). CER and *g*_s_ ESs were significantly positively associated with the leaf N:P ratio of NM plants. The AM-induced stimulation of CER and *g*_s_ increased by 1.4 and 0.2%, respectively, with each unit increase in NM leaf N:P. AM-induced changes in *E* were not related to NM leaf N:P.

We used significance of the test that the regression slope differed from 0 (*p* < 0.05) to interpret the linear regression analyses. Since *R*^2^ analog and *I*^2^ depend on *T*^2^, these statistics cannot be calculated when *T*^2^ is 0 for the analysis. *T*^2^, a measure of between-study or true variance, equates to 0 when *df* exceeds *Q*_total_, i.e., when all variance is statistically defined as within-study variance. In meta-analyses having many studies and large within-study variation, which is often the case in plant physiology, *T*^2^ often equates to 0. For this reason, the two-sided *p*-test of a significant slope is a more reliable and useful metric for evaluating our analyses. It is important to note that while a significant *p*_hetero_ value denotes that true effects vary, the converse is not true. A non-significant *p*_hetero_ value should not be considered evidence that there were no real differences among study effects as there may have been insufficient power to detect them (Borenstein et al., 2009).

For the most part, ESs were more evenly spaced across studies for root colonization and leaf N:P ES than for shoot DW ES and leaf P ES, for each of the three gas exchange parameters (**Figures 3–5**). For the CER and *g*_s_ regressions with the shoot DW and leaf P ES moderators, there were a few very high moderator values with most of the data points appearing clustered within a much narrower range (**Figures 3B,C** and **4B,C**). These high values were not influential points or outliers; they did not diverge from the pattern predicted by the regression. However, removal of the most extreme values from the analysis did in some cases change the slope by a few percent. Where this occurred, removing the extreme values tended to increase the slope. For example, the positive relationship between mycorrhizal influence on CER and mycorrhizal influence on leaf P (**Figure 3C**) shows a 20.2% increase in the CER summary effect per unit increase of the leaf P ES. When the four highest leaf P ES values are removed from the regression (depicted in inset of **Figure 3C**), the slope is changed to reflect a 24.6% increase in the CER summary effect per unit increase of the leaf P ES. The positive relationship between mycorrhizal influence on *g*_s_ and mycorrhizal influence on shoot DW (**Figure 4B**) shows a 9.3% increase in the *g*_s_ summary effect per unit increase of the shoot DW ES. When the 15 highest leaf P ES values are removed from the regression (depicted in inset of **Figure 4B**), the slope is changed to reflect a 12.0% increase in the CER summary effect per unit increase of the leaf P ES. The insets in **Figures 3B,C** and **4B,C** depict the regression over the range in which the majority of data points fell. The purpose of insets is to show the bulk of the data and not to imply that there are outliers. That the extreme points tended to diminish the slope in some instances points to the need for further research.

The CER and *g*_s_ AM/NM response ratios showed a surprisingly good fit to their associated AM/NM leaf N:P response ratios, given the wide range of symbionts and experimental conditions across studies (**Figures 3D** and **4D**). An AM-induced change in leaf P has been unrelated to the AM-induced effects on *E* (**Figure 5C**; **Table 2**). The *E* vs. leaf N:P ES relationship (**Figure 5D**) showed more scatter than this regression for CER and *g*_s_ but was still highly significant (*p* < 0.001; **Table 2**). There were six outliers in the CER and *g*_s_ regressions with NM leaf N:P (given in **Supplementary Material, Data Sheet S1**; Lu et al., 2014), which were excluded from the analysis. No outliers were identified for any of the *E* regressions. The *E* vs. shoot DW ES regression contained high values that caused points to appear bunched and so an inset is provided for the narrower range containing most of the points (**Figure 5B**). The slope of the *E* vs. NM leaf N:P regression was 0 (**Figure 5C**). Removing the four highest values (*Parkia biglobosa*; Osundina, 1995) did not change the slope; it remained 0.

Consistent with the categorical temporal analysis, AM-induced stimulation of CER has been significantly, negatively related to year (**Table 2**; **Figure 6A**). The size of the AM-induced stimulation of CER has declined by an average of 1.7% per year (a very substantial 60% over the 35-year study span). The *E* vs. year of publication regression was not significant (*p* = 0.5, **Table 2**; **Figure 6C**).

#### Multiple Meta-Regression

Among the 1019 studies, 224 had CER data, 172 had *g*_s_ data, and 124 had *E* data, for each of the five regression moderators (publication year was considered separately as a diagnostic). Data for the stress categorical moderator were also provided for these studies. These represent less than half of the studies available for categorical analysis of each gas exchange parameter. Summary statistics for multiple factor regression incorporating the regression moderators are given in **Table 4.** The stress moderator did not explain variation in any of the three gas exchange parameters and so was removed from the analysis. Shoot DW ES ratio was significantly correlated with each of the three gas exchange parameters. Percentage root colonization explained significant variation in CER and *g*_s_. Leaf N:P ES explained significant variation in CER and *E*, and leaf P ES in *g*_s_ and *E*. The *p*_test_ value is a “test of change”: testing if the moderator explains significant further variation after variation explained by other four moderators has been accounted for in the model. Of the three nutritional moderators tested, leaf N:P ES was the only one that predicted CER. Leaf P ES explained significant variation in *g*_s_ and *E*. Leaf N:P of NM plants explained the least amount of variation among the five regression moderators. *R*^2^ analog was 0.0 for the CER and *g*_s_ multiple factor regressions and 100% for the *E* multiple regression, illustrating the limitations of this measure.

**Table 4:** Multi-factor meta-regression of studies having data for each of the five regression moderators.

Moderator	*p*_test_	*p*_test_	*p*_test_
	Carbon exchange rate	Stomatal conductance	Transpiration rate
Root colonization	<0.001	0.020	0.601
Shoot DW ES	0.001	<0.001	<0.001
Leaf P ES	0.741	0.001	0.016
NM leaf N:P	0.540	0.766	0.015
Leaf N:P ES	<0.001	0.155	<0.001

**Gas exchange effect size**	***n***	**Intercept**	***I*^2^ (%)**	***p*_hetero_**	**τ^2^**	***R*^2^ analog (%)**

Carbon exchange rate (CER)	224	0.462	0	0.824	0	-
Stomatal conductance (*g*_s_)	173	0.009	0	0.955	0	-
Transpiration rate (*E*)	124	1.609	6.5	0.281	0.046	100


*CER, *g*_*s*_, and *E* are AM/NM effect sizes; analysis was conducted on log-transformed values (ln*R*) from each study. ES, effect size, denotes that moderator is the study’s AM/NM effect size (response ratio) for shoot dry weight (DW), leaf P concentration, and ratio of leaf N to P concentration. NM leaf N:P is the ratio of leaf N concentration to leaf P concentration in the non-mycorrhizal control plants. *p*_*test*_, “test of change,” *p*-value for moderator explaining significant further variation after the model has been adjusted for the other four moderators; *n*, number of studies; intercept, point at which predicted model line crosses *y*-axis; *I*^*2*^, percent of variation due to real differences among study effects; *p*_*hetero*_, probability that all observed (total) variation is due to sampling error (within-study variation); τ^*2*^, between-studies variance. *R*^*2*^ analog, the true variance explained as a proportion of the total true variance, =*T*^*2*^_*explained*_/*T*^*2*^_*total*_ × 100 where *T*^*2*^_*explained*_ is the true variance explained by the regression and *T*^*2*^_*total*_ is the total true variance.*

## Discussion

Since the first reports of mycorrhizal influence on host water relations, enhanced P nutrition and rate of plant growth have been implicated as mainly or partially responsible for the AM effect (Safir et al., 1971, 1972; Liu et al., 2007). For example, higher *g*_s_ of AM onion (Nelsen and Safir, 1982) and AM sunflower (Koide, 1985) relative to NM controls was attributed to higher P content of AM plants. Fitter (1988) illustrated a close relationship between *g*_s_ and leaf P using AM and NM means from three published articles for three host genera. However, there are also many examples in which CER, *g*_s_, and *E* have been higher in AM plants than in comparably sized NM plants having similar leaf P (e.g., Brown and Bethlenfalvay, 1987; Augé, 1989; Davies et al., 1993; Fay et al., 1996; Ruiz-Lozano et al., 1996). The meta-analysis reveals that when viewed across a literature spanning 35 years and several hundred studies, AM stimulation of CER and *g*_s_ has been significantly linked to AM stimulation of leaf P; part of the reason that AM plants display higher CER and *g*_s_ is that they are larger and/or have more P in their foliage. This does not mean that other mechanisms do not apply or are not more influential in some instances, as much of the variation is not explained by size or nutrition. As others have summarized (e.g., Sánchez-Díaz and Honrubia, 1994; Boomsma and Vyn, 2008; Ruiz-Lozano and Aroca, 2010), it is likely that a symbiosis that affects many aspects of plant physiology can affect carbon and water vapor exchange rates in several ways.

Hoeksema et al. (2010) found that considering relative abundance of P and N better predicted plant response to AM symbiosis than focusing on either element separately. In their meta-regression, AM-induced changes in plant growth were better correlated with tissue N:P ratio than with tissue P or with tissue N. Mycorrhizal benefit is usually greater when plants are P limited (e.g., Propster and Johnson, 2015; Jin et al., 2016), and final tissue N:P of NM plants can serve as an indication of proportionate soil availability of N and P. Values above 16 signify P-limitation and values below 14 signify N-limitation. With gas exchange, we also found that a consideration of leaf N in conjunction with P explained more variation than the customary examination of links with leaf P alone, but in a different way. In our single factor meta-regressions, leaf P ES and leaf N:P of NM plants were of mostly similar value in predicting stimulation of gas exchange by AM symbiosis. An even better predictor of gas exchange response to AM symbiosis was the relative P-limitation of AM and NM plants, signified by the AM/NM leaf N:P response ratio. Leaf N:P of NM plants can be considered a kind of absolute measure of the primary study’s P limitation or abundance, whereas the AM/NM leaf N:P response ratio gives a comparison of a study’s N and P abundance by treatment: a relative measure comparing how much N was contained in leaves of AM vs. NM plants per unit P. As the ratio declined, N was relatively less abundant in AM than in NM leaves, i.e., P limitation was being overcome by the symbiosis. The regression analysis demonstrated that as the symbiosis has increased the relative abundance of P relative to N, its stimulation of host CER and *g*_s_ increased. Drought tends to increase the leaf N:P ratio (inhibit P uptake more than N uptake; He and Dijkstra, 2014), and the increased impact of AM symbiosis on host gas exchange that has been observed during drought (e.g., Augé et al., 2014a) is likely partially related to its ability to assist plants in acquiring P in dry soils.

Attempts to relate physiological responses of the host to percent root colonization by AM fungi may have limited success because percent colonization, while convenient to measure, may not be the most meaningful portrayal of host/fungus interaction (e.g., Allen, 2001). When colonization is determined only at the end of an experiment, a strong correlation with plant response is even less likely (Smith and Read, 2008). Although there are limitations to using one-time colonization measurements to model mycorrhizal activity and physiological response of host plants to the symbiosis, this is the measure that is widely reported in the literature and available for meta-analysis. One advantage of meta-analysis is the greatly increased power generated by regressing over many studies. If past attempts to relate plant responses to percent root colonization have been hampered by insufficient statistical power, meta-analysis may be able to tease these out. The positive correlation of percent root colonization with AM influence on CER, *g*_s_, and *E* may be related to the other regression moderators studied here; more highly infected roots may be better able to scavenge soil P and plants may grow more quickly as a consequence. The positive correlation may also relate to more effective water uptake, modified soil properties, hormonal relations, modulation of drought-induced plant genes, or other mechanisms (Augé, 2001; Ruiz-Lozano et al., 2006; Kaschuk et al., 2009; Xu et al., 2013). In a prior categorical meta-analysis, root colonization explained a significant amount of variation in *g*_s_ (Augé et al., 2014a). When roots of plants in an AM treatment were heavily colonized, the percentage increase in the AM effect on *g*_s_ was 10× greater than the negligible increase observed when roots were more sparsely colonized.

CER, *g*_s_, and *E* are associated measures that often track each other closely (e.g., Bernacchi et al., 2007; Greer, 2012). *g*_s_ and *E* have each been used to monitor stomatal behavior and can track one another particularly closely. *E* is the product of *g*_s_ and vapor pressure deficit, and vapor deficit tends to be similar among treatments within the controlled greenhouse and growth chamber conditions common to most of the studies in the analysis. CER was more markedly affected than *g*_s_ or *E* by AM symbiosis, perhaps related to the much greater carbon sink strength of AM roots relative to non-AM roots (Smith and Read, 2008). Looking at numerous studies, Kaschuk et al. (2009) found that, on average, rhizobial and AM symbioses induced 28 and 14% increases in photosynthetic rates, respectively. With dual symbiosis by both types of microbes, the increase was 51%, about the same as the overall AM-induced average increase in CER computed from the studies in our analysis. As AM fungi may stimulate growth of other microbes in the rhizosphere (e.g., Ames et al., 1984; Marschner and Timonen, 2005), it is likely that host plant roots were supporting both AM fungi and other microbes in many of these 583 studies, which might help explain the higher AM effect on CER. However, the overall summary value of 49% for the AM/NM CER response ratio integrates 35 years, and AM influence on CER has steadily declined over the years that it has been measured by scientists. Aside from the anomalous 1985–1989 year period, the positive AM influence has decreased from a very sizeable 98% for the 1980–1984 period, becoming smaller by approximately 10–20% for each of the 5-year periods between 1990 and 1994 and the present.

Could the lessening of AM influence on CER be related to the steadily increasing atmospheric CO_2_ concentrations that have occurred during this time frame? Perhaps increased CER accompanying increasing atmospheric CO_2_ concentrations (e.g., Curtis and Wang, 1998) has damped differences between AM and NM plants. Alternately, perhaps the down-regulation of photosynthesis that can occur under elevated CO_2_ (as quickly as within 4 weeks of exposure; Sanz-Sáez et al., 2010) tends to muffle the AM effect. AM symbiosis can accelerate photosynthetic acclimation to elevated CO_2_ and promote the down-regulation effect (Goicoechea et al., 2014). *g*_s_ can also diminish with elevated CO_2_ (Lammertsma et al., 2011), yet the size of the AM-induced stimulation of *g*_s_ has not declined during the past 30 years; it has remained fairly stable at 20–30%. Further, rather than encourage it, there is reason to believe that AM symbiosis may alleviate the photosynthetic down-regulation (Fitter et al., 2000; Gavito et al., 2000). At any rate, the elevated CO_2_ treatment in experiments has generally been much larger than the 70 ppm increase in CO_2_ levels in the atmosphere between 1980 and 2014 (Tans and Keeling, 2015).

The promotive impact of AM symbiosis on photosynthetic rate over the 35-year time span has been especially keen when plants were measured during environmental stress. The abiotic stress most often studied in relation to *g*_s_ has been drought, where the mycorrhizal impact has been substantive (Augé et al., 2014a). Mycorrhizal symbiosis has also frequently resulted in higher CER and *g*_s_ in plants exposed to salt stress (Ruiz-Lozano and Aroca, 2010). When drought and salt stress were combined in a stress study with sorghum colonized by *Glomus intraradices* or *Gigaspora margarita*, results were inconsistent and inconclusive (Cho et al., 2006). CER, *g*_s_, and *E* were each markedly enhanced in maize plants exposed to high temperature stress when colonized by *Glomus etunicatum* (Zhu et al., 2011). This symbiont combination also led to increased gas exchange relative to non-AM plants with exposure to low temperature stress (Zhu et al., 2010). AM symbiosis has also helped bolster CER, *g*_s_, and *E* with exposure to ozone stress, in *Phaseolus vulgaris* colonized by *Glomus aggregatum* (Wang et al., 2015). AM symbiosis has resulted in higher gas exchange rates when plants were faced with heavy metal stress, e.g., chromium by *Helianthus annuum* colonized by *G. intraradices* (Davies et al., 2002) and *Ampelopteris prolifera* colonized by *Glomus deserticola* (Singh et al., 2014). In other work, photosynthetic rate remained the same in AM and non-AM poplar plants exposed to Cd, Pb, and Zn (Mrnka et al., 2012). Gas exchange rates of AM and non-AM plants were similar in *G. intraradices*-colonized citrus exposed to flooding stress (Hartmond et al., 1987).

Meta summary effects provide context and direction for future investigations. AM symbiosis has been associated with much more pronounced increases in photosynthesis than in *g*_s_ or *E*. Can this be expected to continue, given the steadily decreasing size of the AM influence on CER over time? Investigators can expect to see smaller AM-induced gas exchange effects if controlling AM and NM treatments for plant size or leaf P. Forecasting AM influence in regard to AM/NM leaf N:P ratio rather than leaf P appears promising. That AM-induced stimulation of each gas exchange parameter was linked to the extent to which root systems were colonized also has important implications, ecologically and experimentally. It may serve as a caution to researchers interested in studying AM influence on gas exchange; they are less likely to observe an influence if roots are only lightly colonized.

## Author Contributions

All authors listed, have made substantial, direct and intellectual contribution to the work, and approved it for publication.

## Conflict of Interest Statement

The authors declare that the research was conducted in the absence of any commercial or financial relationships that could be construed as a potential conflict of interest.

## Supplementary Material

**DATA SHEET S1 | Details on the 1019 studies used in the meta-analyses, including categorical and numerical moderators, sample sizes (*n*), natural log (ln) values of the response ratios with corresponding non-parametric variances, citations, and calculations for synthetic values for multiple time-points.**
